# Far-field optical imaging of topological edge states in zigzag plasmonic chains

**DOI:** 10.1515/nanoph-2021-0648

**Published:** 2022-02-02

**Authors:** Yuto Moritake, Masaaki Ono, Masaya Notomi

**Affiliations:** Department of Physics, Tokyo Institute of Technology, 2-12-1 Ookayama, Meguro-ku, Tokyo 152-8550, Japan; PRESTO, Japan Science and Technology Agency, 4-1-8 Honcho, Kawaguchi, Saitama 332-0012, Japan; NTT Basic Research Laboratories, NTT Corporation, 3-1 Morinosato-Wakamiya, Atsugi-shi 243-0198, Kanagawa, Japan; Nanophotonics Center, NTT Corporation, 3-1, Morinosato-Wakamiya, Atsugi-shi 243-0198, Kanagawa, Japan

**Keywords:** nanofabrication, nanophotonics, optical imaging, plasmonics, topological edge states, topological photonics

## Abstract

Topological photonics mimicking topological insulators has recently attracted considerable attention. The Su–Schrieffer–Heeger (SSH) model, which is a fundamental topological system, has been experimentally demonstrated in many photonic systems owing to its simplicity. In particular, a zigzag chain, which is described by the SSH model, shows intriguing functionality such as polarization-dependent switching of topological edge states. To date, the far-field imaging of topological edge states in plasmonic chains has not been reported because of the constraint imposed by the diffraction limit. In this study, we experimentally observed the photonic topological edge states of zigzag plasmonic chains composed of metal nanodiscs in the optical region through far-field imaging. Using a chain longer than the diffraction limit, light scattering from the two edges of the zigzag chains was resolved. In the case of such a long chain, it was revealed that tiny gaps of several nanometers between the discs, which are difficult to fabricate, are necessary. Therefore, we propose connected chains and investigate the effect of the shape of the connected part, which reveals that similar topological edge states can be obtained even in the connected chains. The polarization dependence of edge-state imaging showed switching of the systems in trivial and topological phases in the same zigzag chain. Far-field observations serve as an easy and effective tool for the investigation and application of photonic topological edge states.

## 1 Introduction

Stimulated by the discovery of topological phases of matter, fields that utilize the topological nature of systems have attracted considerable research attention. In photonics, photonic topological insulators mimicking topological insulators in materials science have been proposed and demonstrated, leading to the emergence of “topological photonics [1–6].” Exotic properties such as robustness against disorder and spin-locked propagation have been reported, which are similar to those of topological insulators.

**Figure 1:**
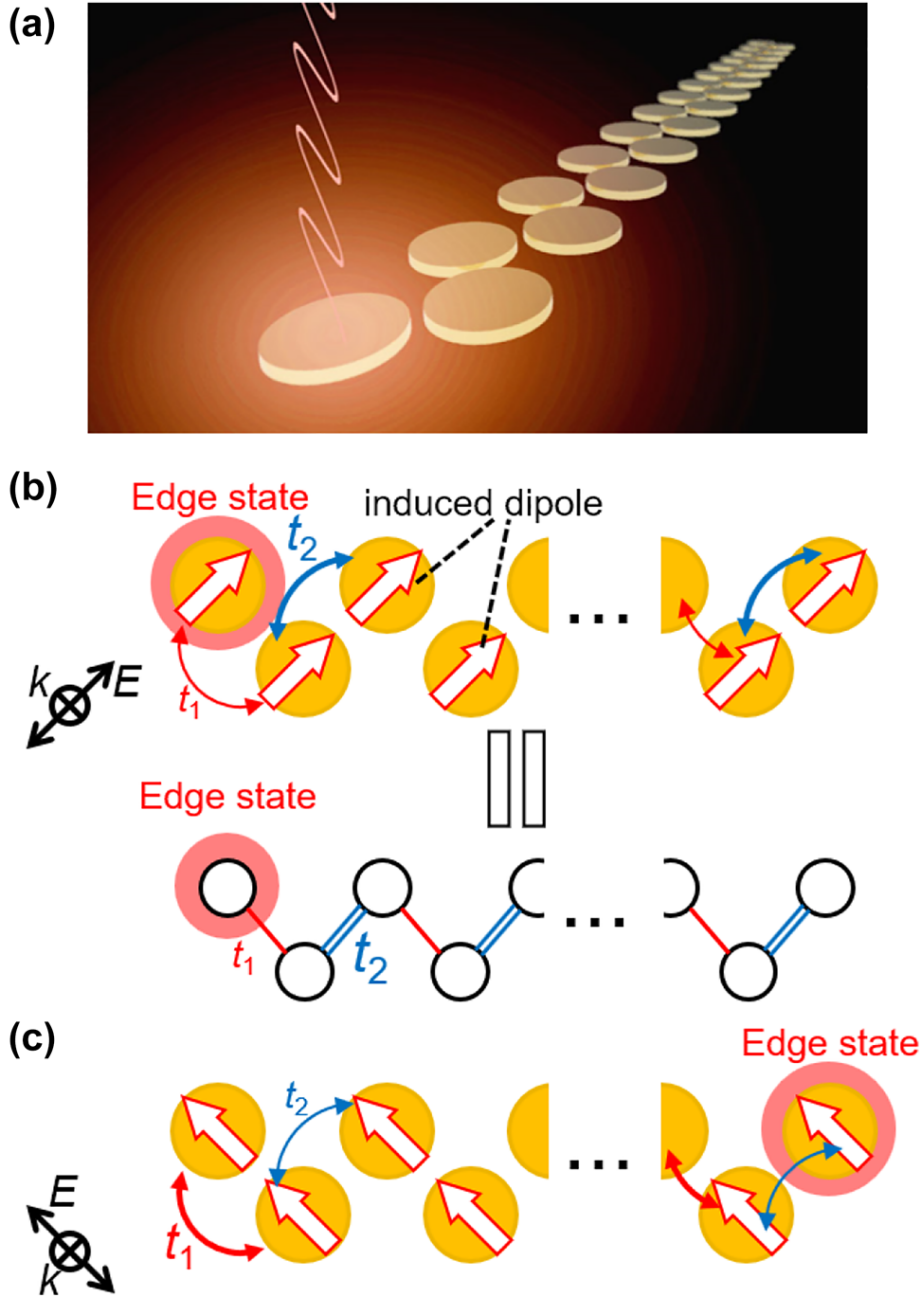
Principles of the topological edge states in the zigzag chain. (a) Photonic topological edge state visualized by a long zigzag chain. Gold nanodiscs are arranged in a zigzag manner. An incident light is impinged normally to the structure surface and localized electromagnetic states originating from the topological edge states are excited. (b), (c) Schematics of the zigzag chain and SSH model. The edge state positions are switched depending on the polarization direction.

Because of its simplicity, the Su–Schrieffer–Heeger (SSH) model [7], which is a fundamental system exhibiting a topological band structure, has been experimentally demonstrated in many photonic systems [8–10]. The SSH model is a tight-binding model with two sites in one unit cell, as shown in Figure 1(b). Depending on the balance between *t*_1_ and *t*_2_, the system exhibits a trivial or topological phase. In the topological phase of photonic SSH systems, topological edge states appear as electromagnetic states localized at the edge of the chain with a frequency inside the bandgap.

One of the intriguing systems described by the SSH model is the one-dimensional zigzag chain [11–19]. The zigzag chain is composed of subwavelength discs arranged in a zigzag manner as shown in Figure 1(b). When light is normally incident on the structure surface, dipole resonance is induced at each disc. The arrangement of dipoles can be manipulated by changing the incident polarization. In the case of the polarization direction as depicted in Figure 1(b), couplings, *t*_1_ and *t*_2_, are different because the dipole–dipole interaction strongly depends on the dipole orientation [20, 21]. In this case, *t*_1_ becomes smaller than *t*_2_, and the system can be regarded as the SSH model in the topological phase, as shown in Figure 1(b), resulting in a topological edge state at the left edge of the chain. The position of the edge state can be easily switched from one edge to the other by changing the incident polarization direction as shown in Figure 1(c), which is a unique property of the zigzag chain.

The visualization of topological edge states is important for investigating the properties of topological photonic systems. In zigzag chains, the edge states are reportedly visualized through near-field and far-field imaging [12, 13, 16], [17], [18]. Although far-field imaging has been reported using zigzag chains composed of dielectric discs [17, 18], it has not been reported for plasmonic chains composed of metal discs. Far-field imaging of the edge states in plasmonic chains is difficult because the chains are not long enough to distinguish the edge state of each edge of the chains. The size of a light-scattering image of the edge state is of the order of the resonant wavelength owing to the diffraction limit; therefore, it is difficult to separate the positions of both edges. To overcome this limitation, the edge states in the plasmonic chains were visualized using a near-field optical microscope, which can obtain an image with a resolution lower than the diffraction limit of light [12].

In this study, to visualize the topological edge states in plasmonic chains through far-field imaging, a chain with a length longer than the resonant wavelength and two spatially separated edges are used, which enables optical imaging of the edge states (Figure 1(a)). The designed long chain is composed of 27 or 28 gold nanodiscs; the longest chain reported so far is composed of 15 dielectric discs [17]. However, in the case of such a long chain, we found that tiny nanometer-scale gaps are necessary to obtain a sufficiently wide frequency bandgap. Because the tiny gaps are difficult to fabricate, we propose the use of connected chains and reveal that similar topological edge states can be obtained. Strong light scattering from the topological edge states in the connected chains was visualized by far-field imaging. The polarization dependence of the edge states shows their polarization-dependent positional change and switching between the systems in the trivial and topological phases. The wavelength dependence of far-field imaging revealed that the edge states can be observed only in the frequency band gap.

## 2 Results and discussion

**Figure 2:**
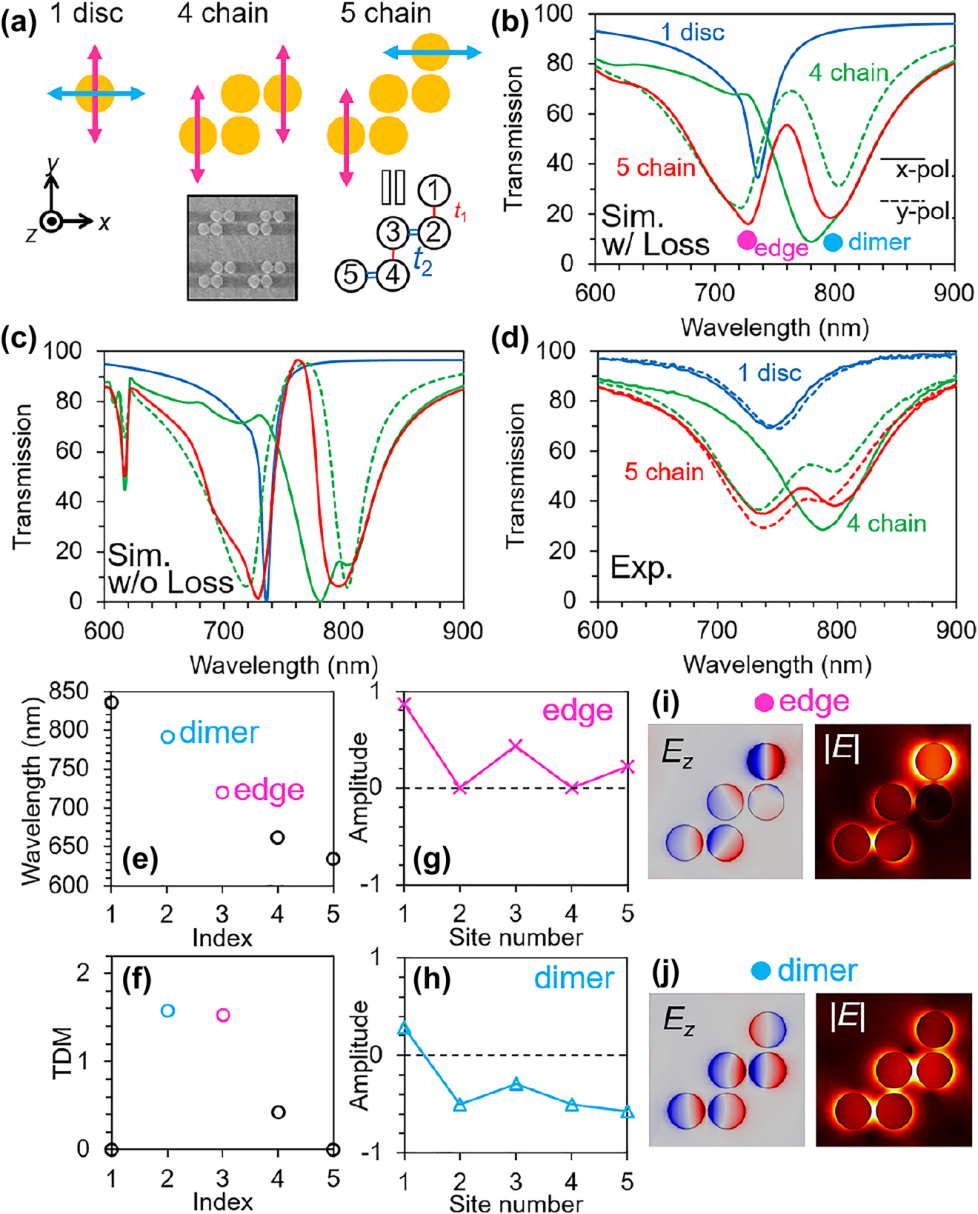
Experimental and numerical analysis on the relatively short chains. (a) Schematics of the 1, 4, and 5 chains and the SEM image of the fabricated sample. The measured disc diameter and gaps are 113.9 and 16.1 nm, respectively. The chains are arrayed periodically in the *x*- and *y*-directions with periods of 500 nm. (b) and (c) Simulated and (d) measured transmission spectra of the 1, 4, and 5 chains with changing incident polarization. Solid and dashed lines correspond to the results for *x*- and *y*-polarizations, respectively. In (c), metallic loss is neglected. (e) Eigenfrequencies (wavelengths) and (f) TDM of the SSH model with 5 sites. Amplitudes at the sites of (g) edge and (h) dimer modes calculated from the SSH model with 5 sites. Simulated electric field distributions at the (i) edge and (j) dimer wavelengths in (b).

First, we numerically and experimentally investigated relatively short zigzag chains composed of 1, 4, and 5 gold nanodiscs (Figure 2(a)) to understand their basic spectral properties. Figure 2(b) shows the simulated transmission spectra of 1, 4, and 5 chains for normal incidence with *x* and *y* polarizations. The simulations in this study were performed using a commercial finite element method solver (COMSOL). The dielectric constant of gold obtained from Ref. [22] was used and the refractive index of the substrate was set to 1.46. The detailed structural parameters were set to the measured values of the fabricated sample listed in the caption of Figure 2.

In the case of one disc (blue lines in Figure 2(b)), only one dipole resonance as a transmission dip and no polarization dependence are observed, as indicated by the blue solid (*x*-pol.) and dashed (*y*-pol.) lines. For the 4 chain (green lines in Figure 2(b)), the excitation dip of the edge mode (Figure 2(i)) at 730 nm is observed only for *y*-polarization, while the dimer mode (Figure 2(j)) at 810 nm is excited at both polarizations. The resonant wavelength of the edge mode is close to that of the single disc, which is the characteristic property of the SSH model. In the case of the 5 chain, the edge and dimer modes are excited at both polarizations as indicated by the red lines in Figure 2(b). Figure 2(d) shows the measured transmission spectra which agree well with the simulated spectra. For the experiments, zigzag gold chains were fabricated on a glass substrate using the conventional lift-off method. The chain patterns were written on a resist through electron beam lithography, followed by the evaporation of a 15 nm-thick gold film.

Figure 2(c) shows the simulated transmission spectra, in which the imaginary parts of the dielectric function of gold are set to 0, which indicates that metallic loss is neglected. By comparing the simulated spectra with (Figure 2(b)) and without (Figure 2(c)) metallic losses, we can discuss radiation and metallic losses separately. Although the metallic loss broaden the resonant dips, the effect is not significant in relation to the radiation loss. Therefore, a dip width of approximately 60 nm is set as the intrinsic limit of the disc system even when dielectric materials are used. The experimental width of the resonance dips (approximately 90 nm) is consistent with that in the simulations with the metallic loss (approximately 100 nm).

Because the 5 chains are composed of five discs (dipoles), there should be five modes in the system. However, only two modes (edge and dimer modes) are observed in the spectra. This can be understood by SSH model analysis. Figure 2(e) shows the eigenfrequencies (wavelengths) derived using a 5×5 matrix describing the SSH model of the zigzag chain. Here, we used the experimental resonant wavelengths of the edge and dimer modes in Figure 2(d) to determine the resonant wavelength of the dipoles and coupling constants between them. The ratio of the coupling constants, *t*_2_/*t*_1_, was assumed to be −2 [13]. Figure 2(f) shows the total dipole moment (TDM) of each mode in Figure 2(e). The TDM is the sum of the amplitudes of all the sites of the system, which is proportional to the radiation loss, and determines whether the mode can be excited by normal incidence. Here, the amplitude means the components of the eigen-vectors of the matrix. The mode with a large TDM can be excited by normal incidence and appears as a dip in the transmission spectra, whereas a small TDM mode is unexcitable. From Figure 2(f), the modes of indices 2 and 3 corresponding to the dimer and edge modes, respectively, have large TDMs, which is the reason why these modes are observed in the transmission spectra. As shown in Figure 2(i) and (j), *E*_*z*_ distributions, which indicate the charge distributions, agree well with the amplitude distributions in Figure 2(g) and (h). In the case of the 5 chain, the modes can be easily separated in the spectra even when the gaps between the discs are approximately 16 nm because the number of modes is small and the modes are sparse in the frequency region. This situation changes in the case of a long chain, as discussed later.

**Figure 3:**
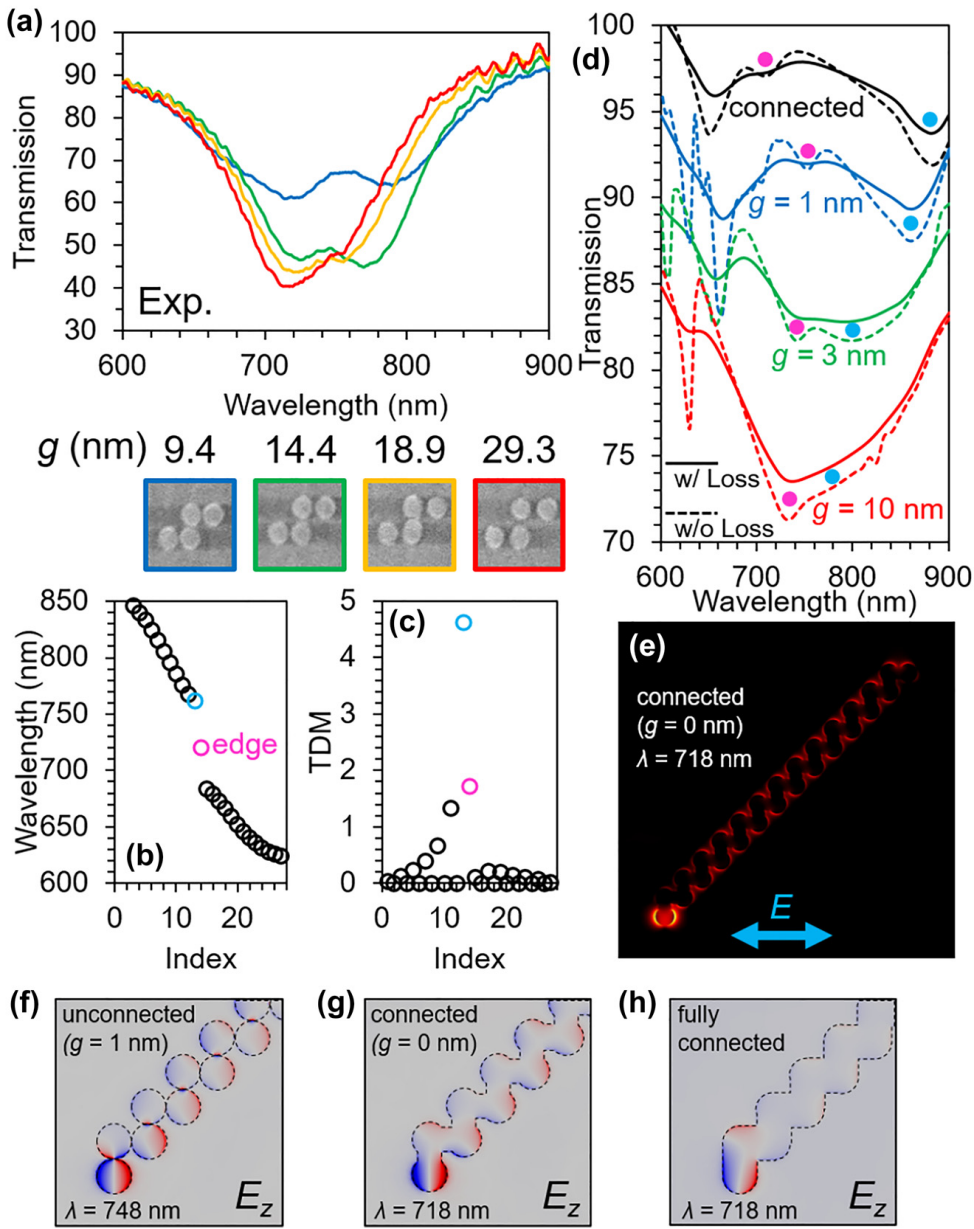
Experimental and numerical analysis on the long chains. (a) Measured transmission spectra for the 5 chain with different gaps. (b) Eigenfrequencies (wavelengths) and (c) TDM of the SSH model with 27 sites. (d) Simulated transmission spectra for the 27 chain with different gaps. Solid and dashed lines correspond to the results with and without metallic loss, respectively. (e) Simulated electric fields of the edge states in the 27 chain at the wavelength of 718 nm. (f)–(h) Simulated *E*_*z*_ distributions of the edge states for the (f) unconnected (*g* = 1 nm), (g) connected, and (h) fully connected chains.

Next, we discuss the influence of the gap between the discs on the frequency bandgap. Figure 3(a) shows the transmission spectra of the 5 chains with changing gaps *g*. Blue, green, orange, and red lines correspond to the results for *g* = 9.4, 14.4, 18.9, and 29.3 nm, respectively. As the gap increases, the frequency difference between the edge and dimer modes narrows, which suggests that the coupling between the dipoles induced at the discs reduces.

Figure 3(b) and (c) show the eigenfrequencies (wavelengths) and TDM derived from a 27×27 matrix describing the 27 chain system. Here, we used the experimental resonant wavelengths of the edge and dimer modes in the case of *g* = 9.4 nm in Figure 3(a), which is the smallest gap that we successfully fabricated in the experiments, to determine the resonant wavelength of the dipoles and coupling constants between them. In contrast to the 5 chain system, the bulk modes form continuous bands, and the wavelength difference between the edge and bulk modes is less. The frequency bandgap is almost the same as that predicted by the SSH model. The edge mode has a TDM similar to that of the 5 chain case. Importantly, the TDM of the mode at the band edge of the continuous band, depicted by the light blue color, is larger than that of the edge mode, which indicates that the mode has a large radiation loss and is observed to have a broad transmission dip. This is undesirable because the wavelength difference between this mode and the edge mode is only approximately 40 nm, and these modes must overlap each other in the spectra. Simulations for the 27 chain with a gap of 10 nm (red line in Figure 3(d)) show a large overlap between them in the spectra. As shown in Figure 3(d), a small gap of several nanometers is necessary to achieve a sufficiently wide bandgap to distinguish these modes. However, such a small gap is difficult to fabricate experimentally. We note that this problem will occur even when the chains are composed of dielectric materials because a large overlap is observed even when the metallic loss is negligible (dashed lines). If the arranged angle in the zigzag chain deviates from 90°, the coupling contrast between *t*_1_ and *t*_2_ gets smaller, which leads shrinking of the frequency bandgap [16], which will make the far-field imaging of the edge states more difficult.

To avoid the problem, we propose connected zigzag chains for the observation of far-field images of topological edge states. The black line in Figure 3(d) shows the transmission spectra of the connected (*g* = 0 nm) 27 chain. Although the resonant wavelength of the edge state shifts slightly to the blue side, the resonance dip is separated from the other modes, and a similar electric field distribution is obtained, as shown in Figure 3(e). Figure 3(f), (g), and (h) show *E*_*z*_ distributions of the edge states for the unconnected (*g* = 1 nm), connected, and fully connected chains, respectively. In the cases of the unconnected (Figure 3(f)) and connected chains (Figure 3(g)), the dipole modes at the edge site of the chains are observed, and overall fields are similar each other. On the other hand, the dipole mode is no longer observed for the fully connected chain (Figure 3(h)). Therefore, the shape of the connected parts is important to achieve similar properties of the edge states in the zigzag chain. Simulations revealed that the edge mode in the connected zigzag chain exhibits the same polarization dependence, which will be proven by experiments later.

**Figure 4:**
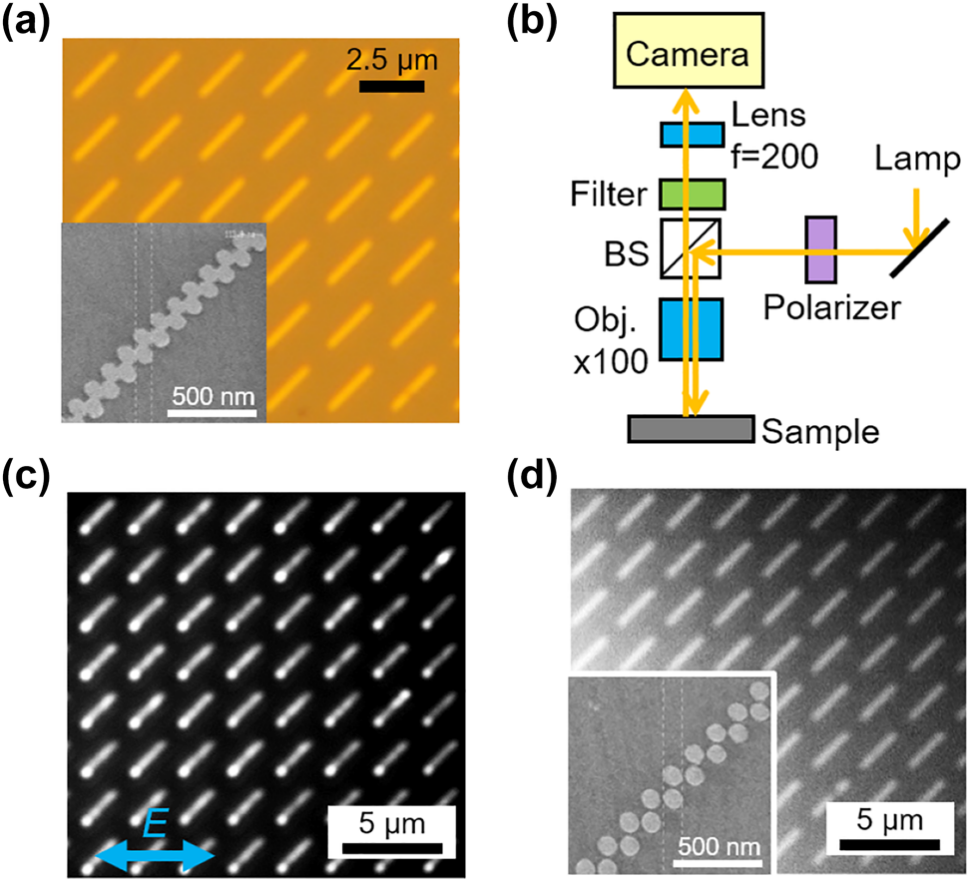
Experimental images of the fabricated chains. (a) Microscopy and SEM images of the fabricated array of the 27 chains. The measured disc diameter is 117 nm. The chains are arrayed periodically in *x*- and *y*-directions with periods of 1500 nm. (b) Experimental setup for far-field imaging. (c) Far-field image of edge states in the 27 chain sample shown in (a) for *x*-polarization. (d) The far-field image of the 27 chain with the gap between discs shown in the SEM image (inset). The measured gap is 17 nm.

We experimentally investigated a chain composed of 27 discs, which contains a larger number of discs than that reported in previous studies [11–18]. The optical microscope and scanning electron microscopy (SEM) images of the connected chains are presented in Figure 4(a). The detailed structural parameters are listed in the caption of Figure 4. The experimental setup for far-field imaging is shown in Figure 4(b). The setup is a typical microscope setup, despite the use of a high-magnification objective lens (×100) and a high-sensitivity camera. Band-pass filters with a center wavelength of 750 nm and FWHM of 10 nm were used to select the excitation wavelength. Figure 4(c) shows strong light scattering from the edge of the chain, which originates from the excitation of the topological edge states. Light scattering from the edge state were observed in many chains, despite the inhomogeneity of the structures due to finite fabrication errors, implies robustness to structural imperfection as long as the edge mode is spectrally separated from other modes. In the case of the sample with gaps of approximately 17 nm (Figure 4(d)), the strong light scattering from the edge, as shown in Figure 4(c), is not observed because of the spectral overlapping of the bulk and edge modes, as discussed above. Near-field imaging techniques can visualize electromagnetic fields with subwavelength resolution; however, the emission properties of the edge states cannot be obtained. In contrast, because far-field imaging contains information about light emission, the edge states in the fabricated plasmonic chains, visualized by far-field imaging, find applications that utilize luminescence [18] and nonlinear effects [17].

**Figure 5:**
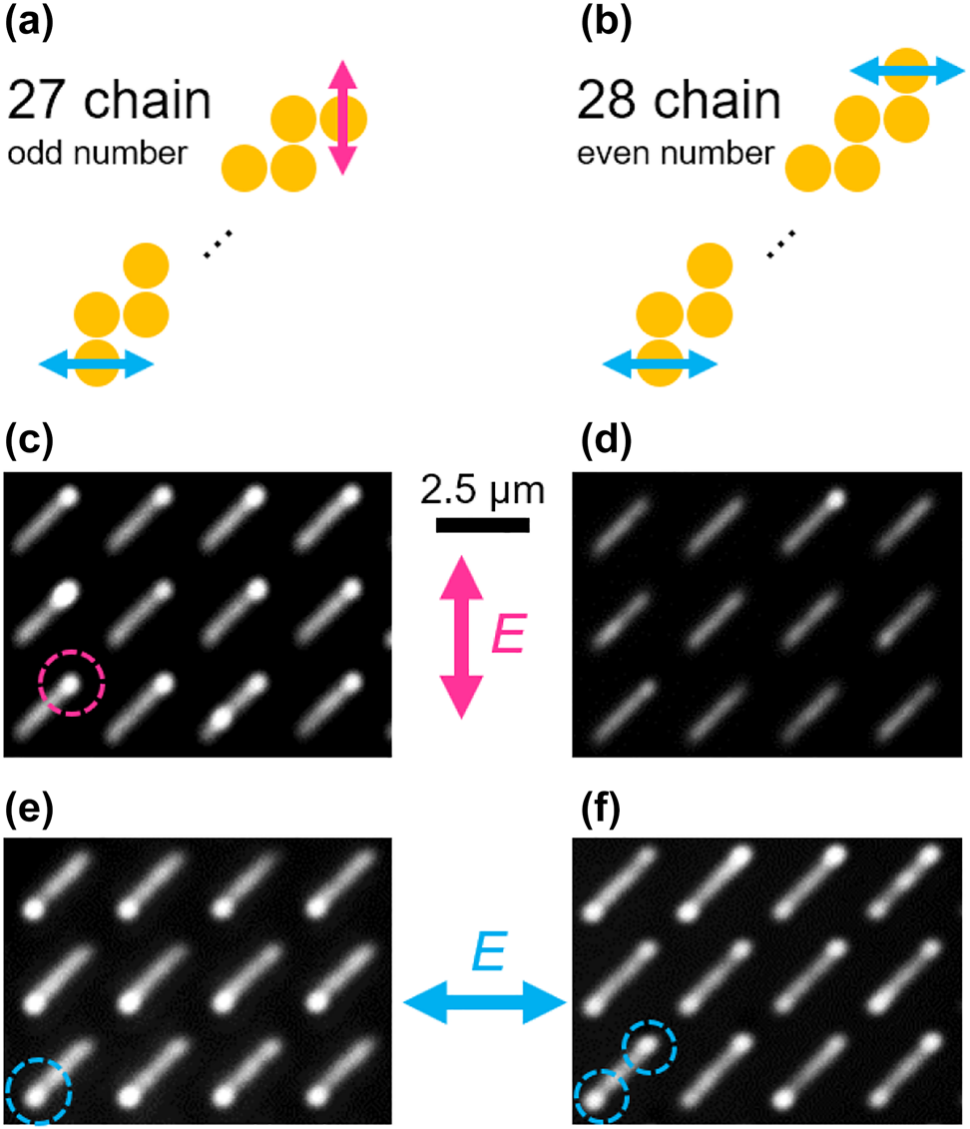
Polarization dependence of the edge states. Schematics of polarization dependence of the edge states in the (a) 27 chains and (b) 28 chains. Measured far-field images of the array of the (c), (e) 27 chains and (d), (f) 28 chains for the (c), (d) *y*-polarization and (e), (f) *x*-polarization.

If the coupling constants, *t*_1_, *t*_2_, are not equal for a finite chain with odd number of sites, described by the SSH model, the edge state exists on either the right or left edge. Therefore, in the case of the 27 (odd number) chain, the edge states exist for both *x*- and *y*-polarizations, and the polarization determines the position of the edge states as shown in Figure 5(a). In contrast, in the case of the 28 (even number) chain, the existence of the edge states is determined by the phase of the system. For the *y*-polarization, intra-coupling *t*_1_ is larger than inter-coupling *t*_2_, which indicates that the system is in a trivial phase, and there are no edge states. In contrast, for the *x*-polarization, *t*_1_ < *t*_2_, the edge states appear at both edges of the chain. A clear polarization dependence of the edge states, as discussed above, is observed in the images as shown in Figure 5(c)–(f). In the case of the 27 (odd number) chain, the positions of the edge states are switched between the upper right and lower left, depending on the polarization (Figure 5(c) and (e)). As shown in Figure 5(d) and (f), the edge states are observed at both edges for the *x*-polarization, whereas there is no scattering from the edges for the *y*-polarization in the case of the 28 (even number) chain, which indicates switching between the systems in trivial and topological phases by changing the polarization direction.

**Figure 6:**
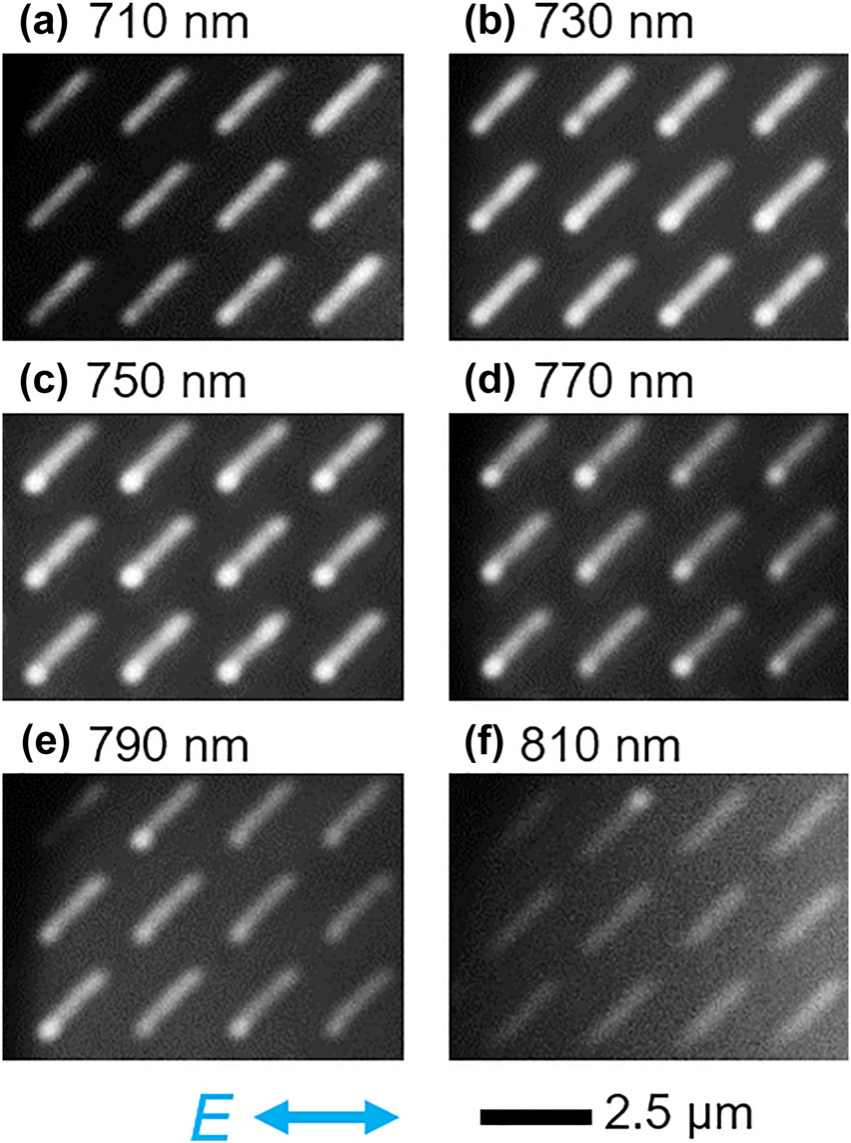
Wavelength dependence of the edge states in the 27 chains for the *x*-polarization. The center wavelengths of the filters are (a) 710, (b) 730, (c) 750, (d) 770, (e) 790, and (f) 810 nm.

Figure 6 shows wavelength dependence of the 27 chain for *x*-polarization incidence. To observe wavelength dependence, the center wavelengths of the filters were changed from 710 to 810 nm. The edge states are clearly observed in the case of 730, 750, and 770 nm and, are not obvious for 710 and 810 nm, which implies that 710 and 810 nm are outside of the frequency band gap.

## 3 Conclusions

We used the long-connected zigzag chains to observe the photonic topological edge states of the zigzag plasmonic chains, composed of metal nanodiscs in the optical region through far-field imaging. We revealed that a small gap is necessary to spectrally separate the edge and bulk modes. By using a chain longer than the diffraction limit, the two edges of the zigzag chain were resolved, and the topological edge states were visualized using far-field imaging. The polarization dependence of the edge state imaging showed the polarization-dependent position changes and switching of the systems in trivial and topological phases in the same zigzag chain. Wavelength dependence revealed that the edge states can be observed only in the frequency bandgap. Far-field observations serve an easy and effective tool for the investigation and application of photonic topological edge states.
